# A simple method for finding a protein’s ligand-binding pockets

**DOI:** 10.1186/1472-6807-14-18

**Published:** 2014-07-19

**Authors:** Seyed Majid Saberi Fathi, Jack A Tuszynski

**Affiliations:** 1 Department of Physics, Ferdowsi University of Mashhad, Mashhad, Iran; 2 Department of Physics, University of Alberta, Edmonton, Alberta, Canada

**Keywords:** Protein structure, Ligand-binding pockets, Computational methods

## Abstract

**Background:**

This paper provides a simple and rapid method for a protein-clustering strategy. The basic idea implemented here is to use computational geometry methods to predict and characterize ligand-binding pockets of a given protein structure. In addition to geometrical characteristics of the protein structure, we consider some simple biochemical properties that help recognize the best candidates for pockets in a protein’s active site.

**Results:**

Our results are shown to produce good agreement with known empirical results.

**Conclusions:**

The method presented in this paper is a low-cost rapid computational method that could be used to classify proteins and other biomolecules, and furthermore could be useful in reducing the cost and time of drug discovery.

## Background

Essential information regarding protein function is generally dependent on the protein’s tertiary structure. This includes the enzymatic function of a protein, and also the binding of ligands, such as small molecule inhibitors [1]. Methods developed for predicting an enzymatic function of a protein by identifying catalytic residues include: finding local characteristics of functional residues [2,3], applying known templates of active sites [4,5] or identifying the surface shape of active sites [6-10].

In order to predict ligand binding (sites, poses and affinities), we first need to determine a 3-dimensional structure of the protein in question, which can be done using several experimental or computational methods [11,12]. Structure-based pocket prediction employs geometrical algorithms or probes mapping/docking algorithms [13]. Comparing these two kinds of methods, it can be said that the geometrical algorithms have low computational costs in contrast to the mapping/docking and scoring of molecular fragments, but the latter algorithms have a greater physical meaning. Geometrical algorithms analyze protein surfaces, and once a structure has been determined, a number of algorithms may be used to predict binding pockets on the protein surface [14-19]. One such example, SURFNET [15], fits spheres into the spaces between protein atoms and finds gap regions. The results obtained this way correspond to the cavities and keys of a given protein. An algorithm based on geometric hashing called VISGRID [20] uses the visibility of constituent atoms to identify cavities. “Active site points” are identified by PASS [19]. In this method the protein surface is coated with a layer of spherical probes, then those that clash with the protein or which are not sufficiently buried are filtered out. The active site points are identified from the final probes. Another method is LIGSITE [14,21], which is an improvement of the POCKET algorithm [22]. This algorithm puts protein-occupied space in a grid and identifies clefts by scanning areas that are enclosed on both sides by the protein’s atoms. An alpha-shape algorithm is used by CAST [17] and APROPOS [18]. DRUGSITE [13] and POCKET-FINDER [23], in addition to the protein’s shape, also consider physicochemical properties for identification of ligand binding pockets. Further geometrical algorithms are TRAVEL DEPTH [24], VOIDOO [25], and CAVITY SEARCH [26]. QSITEFINDER [16] uses interaction energy computation between the protein and a van der Waals probe to find favorable binding sites. Some methods using mapping/docking and scoring of molecular fragment concepts are described by Dennis et al. [27], Kortvelyesi et al. [28], Ruppert et al. [29], and Verdonk et al. [30]. There are also several docking based methods that use ligands to probe the proteins for binding sites [31-34].

Computer-aided drug design often applies protein–ligand docking methods, most commonly structure-based methods. These methods provide support to the rational design and optimization of novel drug candidates [35]. Many structure-based protein–ligand docking methods have been reported in the literature [36-41]. These methods generally rely on first identifying a ligand-binding pocket in the protein structure.

Finding a comprehensive, fast and automated method that can accurately predict ligand-binding pockets on protein surfaces is a major challenge in virtual screening biophysics. This goal leads us to introduce a new method for finding putative ligand-binding pockets on a protein surface, and for identifying the most important characteristics of these pockets: surface area, volume, and potential interacting atoms. This information could be used to cluster protein pockets into similarity classes, and could be a valuable resource leading to a significant decrease in the cost and time expended in the drug discovery process.

The method we present in this paper is based on computational geometry and voxelization concepts. In this method we do not use Delaunay tessellation, the vision criterion, or fitting spheres between atoms, in contrast to some of the methods mentioned above. The CASTp method has used the Delaunay triangulation and the Voronoi concepts to find putative pockets and voids. This method triangulates the surface atoms and clusters triangles by merging small triangles to neighboring large triangles [14,17]. In our work we simply use the convex hull concept and generate a pocket by a grid box formed by the extreme points of a triangle. Then, we consider only the atoms closest to the triangle in the formed pocket. The distance to the convex hull is used for choosing the surface atoms. Thus, our method is not iterative and does not require a flow through all points, hence the computational cost is relatively low. We also take only a given number of empty voxel neighbors for each atom. Voxelization of space for finding putative pockets does not have an essential role for finding surface atoms, unlike VISGRID or grid-based methods, which are based on searching for empty voxels in different directions. We also use voxelization for finding the positions of possible ligands and also to determine physical properties of the pockets.

Comparative modeling methods use fold assignment and template selection for comparing the target protein to a set of proteins with known structures and to search for homologous proteins that have approximately similar structures. Some of these methods are BLAST [42,43], PSI-BLAST [44] and HHpred [45]. I-TASSER [46] is a composite approach of comparative modeling and threading methods [47]. A summary of comparative modeling is given by [48]. In our method we also consider *some* biochemical properties of the protein’s atoms and residues as is explained below. Hence, the proposed method is not purely geometrical. We demonstrate that the results obtained using this method are in good agreement with empirically known results. Hence developing it further may offer even more accurate and reliable results.

## Methods

**Figure 1:**
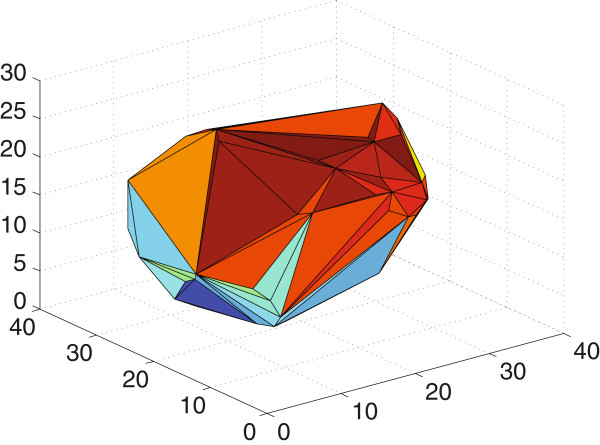
The 3D polyhedron (convex hull) for the PDB:1ABT structure.

**Figure 2:**
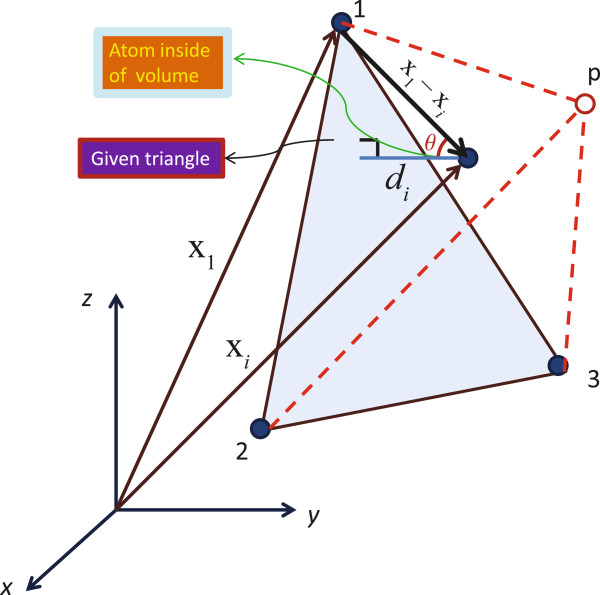
**A given triangle on the convex hull for the PDB:1ABT structure.** The three vertices are labeled as 1, 2, and 3. The point p is determined by the extreme values of *x*, *y*, and *z* of these three vertices. The distance of atom *i* to the triangle is obtained as follows: first obtain the normal vector to the triangle, N, **N =** **(x**_**2**_ **−** **x**_**1**_**)** **×** **(x**_**3**_ **−** **x**_**1**_**)**, where x_1_, *x*_2_, and x_3_ are the vectors from the origin of the systems of Cartesian coordinates to the three vertices. Then, calculate the angle between the normal vector and the line passing through atom *i* and one of the vertices of this triangle using the following relation: $\cos\theta =\frac{\left(x_{1}-x_{i}\right)\cdot N}{\left|x_{1}-x_{i}\right|\left|N\right|}$, Finally, we compute this distance by ***d***_***i***_ = **|x**_**1**_ **−** **x**_***i***_**|** cos *θ*, where x_*i*_ is a vector joining the origin and a given point in this volume.

**Figure 3:**
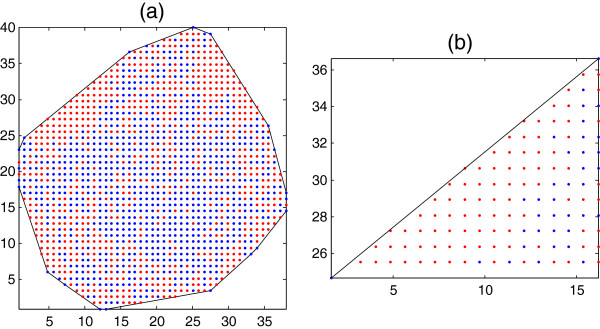
**The steps of the algorithm illustrated (in 2D for clarity) using the PDB:1ABT structure.** The red dots represent empty voxels and the blue dots are voxels containing protein atoms. The atom positions have been averaged on the *z*-axis. **(a)** A convex hull enclosing the protein atoms is generated. **(b)** A line (a triangle in 3D) on the surface of the hull is selected. Inside of convex hull part of a given pocket is shown.

We first voxelize the volume of a box defined to contain the extreme points of the protein’s atomic positions. Then, we use the convex hull concept to obtain the smallest convex polyhedron containing all of the protein’s atoms. In 3-dimensional space, a convex hull surface is formed by triangles, as shown in Figure 1. In the present context, each of these triangles can define a pocket, as illustrated in Figure 2. To define a specific pocket, we consider the volume generated by the extreme positions of the triangle vertices as follows: each triangle contains three vertex points,

$$r_{i}\equiv \left(x_{i},y_{i},z_{i}\right),\left(i=1,2,3\right),$$

which we should consider as

$$\left(\mathrm{extreme}\left(x_{i}\right),\mathrm{extreme}\left(y_{i}\right),\mathrm{extreme}\left(z_{i}\right)\right),$$

where “extreme” indicates either a minimum or a maximum value. Figure 2 shows a given triangle on a convex hull. We have made the grids with a length of 1 Å between ([x_min_ : x_max_], ([y_min_ : y_max_], ([z_min_ : z_max_]) in each axis.Normally, some parts of this rectangular cube are out of the convex hull, but we do not concern ourselves with them because they will be eliminated by another criterion, namely to keep only a given number of empty voxels near each protein atom in a pocket. Figure 3 shows only the inside of a convex hull part of a pocket in 2-dimensions and its grid is shown by points (the blue color represents the atoms and the red represents the empty grid points).

**Figure 4:**
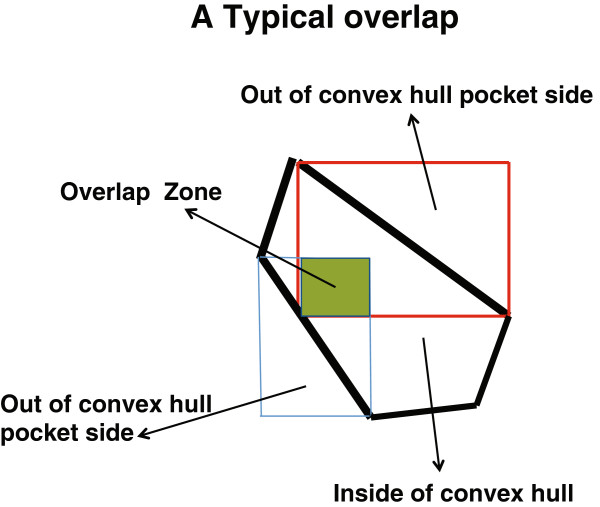
Schematic illustration of the overlap between two pockets.

Then, we obtain the voxels, which are contained within this generated volume, and separate the voxels into those that contain protein atoms and those which do not. Next, we identify the nearest empty voxels with respect to these protein atoms. These empty voxels give us the possible positions of ligand atoms for this particular protein pocket. At this step, we have found a large number of “pocket” envelopes and all the atoms belonging to these pockets are the “protein’s surface atoms”.In some cases, the entire space (or part thereof) under a triangle is common with another space so we say that these spaces overlap with each other. The overlap is defined by the number of atoms in common between the two pockets divided by the total number of atoms in a pocket, which means the overlap is also dependent on the size of a pocket, so that the overlap between two pockets is not symmetric. Figure 4 shows the overlap between two pockets in 2-dimensional space. As we can see in this figure, the overlap size of the common site (determined by the number of common atoms) divided by the size of the pocket (the total number of atoms in the pocket) for each pocket is different.

If all atoms contained in a set of the pocket atoms exist in the other pocket, it has an overlap of 100%. However, the second pocket may have more atoms than the first one, i.e. it has all atoms of the first pocket plus other atoms. For example, the overlap between pockets #1 and #2 might be 100% while the overlap between pockets #2 and #1 is only 50%, because the number of atoms in pocket #2 is twice as large as the number of atoms in pocket #1, and all atoms belonging to pocket #1 are also contained in pocket #2, but only a half of the atoms in pocket #2 are also in pocket #1. Accumulating all pockets with a given overlap between them as new pockets is the next step.

The final step is related to biochemical and physical criteria such as hydrophobicity, hydrogen bonding, ionic and van der Waals interactions, and also the depth, surface area and volume comparisons between a given pocket and a ligand. By using biochemical conditions, we can find which atoms and which corresponding residues could potentially participate in an interaction with the ligand’s atoms. Tables 1 and 2 propose a set of simple biochemical conditions. It should be noted that to find an active site, more accurate conditions should lead to more accurate results. In this step we can also compute the size of pockets.

**Table 1:** **Main biochemical interactions of atoms and residues in the proteins**[49,51,52]

**Residue Name**	**Interactions**
THR	**HBD**: OG1 (OH)
SER	**HBD**: OG (OH)
GLN	**HBA**: NE2 (NH_2_)
ASN	**HBA**: ND2 (NH_2_)
TYR	**HBA**: O – **HBD**: N, OH – **CR**: CE1, CE2, CD1, CD2, CZ, CG
CYS	**Sul**: SG (SH)
MET	**vdW**: CE (CH_3_) – **Sul**: SD (S-CH_3_)
ALA	**vdW**: CB (CH_3_)
PRO	**vdW**: CB (CH_2_), CD (CH_2_), CG (CH_2_)
LEU	**vdW**: CD1 (CH_3_), CD2 (CH_3_), CG (CH)
VAL	**vdW**: CG1 (CH_3_), CG2 (CH_3_), CB (CH)
ILE	**vdW**: CD1 (CH_3_)
ASP	**HBA**: OD1(C = O) – **Ion(−)**: OD2 (OH)
GLU	**HBA**: OE1(C = O) – **Ion(−)**: OE2 (OH)
LYS	**Ion(+)**: NZ (NH_3_)
ARG	**Ion(+)**: NH1 (NH_2_) *trans*, NH2 (NH_2_) *cis*
HIS	**Ion(+)**: NE1 (NH_2_) *trans*, NE2 (NH_2_) *cis* – **CR**: CD1, CE1, CD2, CE2, CG
PHE	**CR**: CG, CD1, CE1, CZ, CE2, CD2
TRP	**HBD**: NE1 (NH) – **CR**: CD2, CE2, CZ2, CH2, CZ3, CE3
TYR	**HBD**: OH – **CR**: CD1, CE1, CE2, CZ, CD2, CG
GLY	No participation

*Abbreviations used:HBA*: Hydrogen bond acceptor, *HBD*: Hydrogen bond donor, *vdW*: van der Waals interaction, *Ion*: Ionic interaction, *Sul*: Sulfur interaction.

**Table 2:** Ligand biochemistry

**C-Ring in ligand**	**C or N atoms in ligand recognizing by connection information in the PDB**
Unprotonated atoms in ligand	1) O has a connection with N, P or Zn
	2) O only has a connection with C
Protonated atoms in ligand	1) Ca
	2) N has only two connection with C

The bond list is given in the PDB file CONECT lines.

A detailed description of the algorithm is given in the following:

The algorithm

1. Input protein atom position data, and define a box by using the extreme positions of the atoms.

2. Voxelize the box by considering the voxel with 1 Å in length, width and height.

3. Compute the convex hull surrounding the protein atoms and obtain the volume of the convex hull and the surface area of atoms.

4. Separate empty voxels (possible ligand atom positions) from voxels filled by the protein atoms in the convex hull.

5. Define the pockets by the volume generated by the vertices of each triangle on the convex hull.

6. Compute the overlap between two neighboring pockets and assemble the pockets with an overlap greater than a minimum value (reconstruct new pockets).

7. Find the physical properties of the pockets such as depth, surface and volume.

8. Find the residues corresponding to the pocket atoms.

9. Assess the biochemical conditions [49,50] as introduced in Table 1 (we use the IUPAC nomenclature [51] and the PDB format [52]). In this step we can find the atoms and residues that participate in the potential active site.

10. Compare physical and biochemical properties between ligand atoms (Table 2) and the atoms of a given pocket, such as: the size of pockets (depth, surface and volume) with ligand size, the number of hydrogen donor/acceptor atoms, possible rings, or van der Waals interactions, etc.

Supplementary steps to compare our results with known active sites

11. Compute the number of correct residues predicted in each pocket of the unliganded protein and divide it by the number of residues in an “active site” of the liganded protein as reported in the PDB, i.e.

$$\begin{array}{l} \mathrm{cf}=\mathrm{correct}\;\mathrm{fraction}\\ \;=\frac{\mathrm{number}\;\mathrm{of}\;\mathrm{correct}\;\mathrm{residues}\;\mathrm{predicted}\;\mathrm{in}\;\mathrm{pocket}}{\mathrm{number}\;\mathrm{of}\;\mathrm{residues}\;\mathrm{in}\;\mathrm{active}\;\mathrm{site}} \end{array}$$

**Figure 5:**
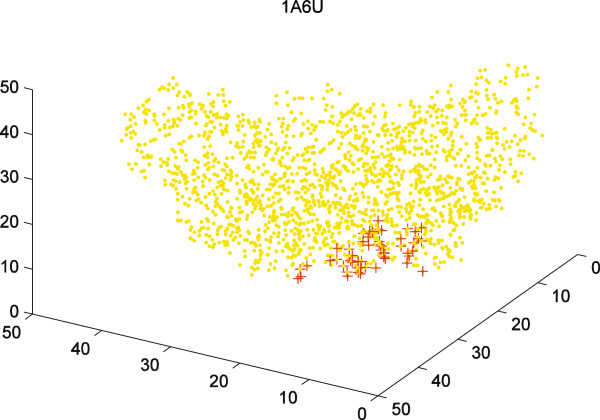
**Three dimensional structural representation of 1A6U.** The atoms are shown with yellow dots and the surface atoms of a given pocket are shown with red crosses.

12. *Optional step.* Compute the minimum distance between the ligand atoms and each residue atoms in the pocket. Then, filter residues of a pocket with the minimum distance greater than the given values, for example 3.50 Å.In Figure 3, we illustrate these steps in 2-dimensional space for better clarity. Here, we need to use a line instead of a triangle to define a pocket. Figure 5 uses the example of the protein labeled 1A6U in the PDB. It shows 3-dimensional atomic positions of the protein and the atoms that belong to a pocket.

## Results and discussion

In reality, the geometrical criteria give initial information about physical properties for the possible protein-protein or protein-ligand docking, determining shapes, sizes, etc. For docking to occur, the recognized geometrical protein pocket should be a protein’s active site. Finding active sites is very complicated for both *in vitro* and *in silico* methods. There are many computer programs that find active sites [13-23] but they have high computational cost associated with them and also they do not typically determine physical properties of the active site which means that we need to find a ligand in spite of lacking some important information. Therefore, it is imperative to use mixed geometrical and biochemical methods to find possible pockets in a protein. This paper has introduced a method to find protein pockets with a higher probability of interactions than based on exclusively biochemical methods. This method offers a speed-up of the drug discovery process by allowing clustering of both the protein pockets and ligands.

We first demonstrate our method by describing an example, namely a pair of unliganded and liganded proteins, 1A6U and 1A6W. We have used only non-water atoms of 1A6U to find its pockets. These pockets are reported in Table 3. To verify these results, we check the SITE REMARK lines for the PDB file of its liganded pair structure, i.e. 1A6W, and we compare the residues of each active sites of the PDB file 1A6W with the residues obtained in each computed pocket. Then, we obtain the *cf* –value for each active site. The last two columns of Table 3 report these values.

**Table 3:** Pockets and their characteristics recognized by our method for 1A6U protein atoms

Pocket Num.^*^	**Num. of Atoms**	**Num. of Empty voxels**	**Surface of Pocket**	**Depth of Pocket**	NoA^** ^**HA**^***a ***^**Bonds**	**NoA HD**^***b ***^**Bonds**	NoA vdW^*** ^**Bonds**	**NoA Ionic Bonds**	**NoA Sulfur Bonds**	**NoA**	***cf***	***cf***
										**C-Ring**	**of the 1**^**st **^**AS, HAP**^***c***^	**of the 2**^**nd **^**AS, AC1**^***c***^
1	63	401	116.25	28.40	5	8	0	1	0	20	0.31	0.33
5	80	481	21.83	38.66	2	3	10	2	0	2	0	0
18	101	648	187.27	25.83	5	7	6	2	0	14	0.12	0.11
19	67	411	84.36	19.35	1	2	5	0	0	2	0	0
38	44	266	138.90	20.63	1	4	1	0	0	6	0	0
39	85	499	82.58	28.26	3	5	2	0	0	14	0.31	0.22
40	21	127	77.97	14.53	2	3	0	0	0	4	0.06	0
58	118	765	340.90	29.83	5	4	7	3	0	3	0	0
59	86	529	253.20	26.72	4	4	4	2	0	6	0.06	0
85	226	1360	370.14	36.18	7	7	26	3	1	27	0	0
89	21	141	212.35	21.47	0	1	4	1	0	4	0	0
90	92	573	293.28	28.54	4	2	15	2	0	11	0	0
112	44	241	36.33	27.39	1	2	1	0	0	6	0.06	0
117	38	215	76.66	17.42	1	3	0	0	0	8	0	0
137	15	99	127.57	17.53	2	4	0	0	0	3	0.25	0.33
143	55	354	259.10	24.24	4	8	0	1	0	20	0.43	0.55

^*^Pocket number indicates the number in the protein’s atomic positions convex hull surface rows, and they correspond to three vertices of triangles.

^**^NoA means the number of atoms.

^***^vdW means van der Waals.

^*a*^HA means hydrogen bond acceptor.

^*b*^HD means hydrogen bond donor.

^*c*^These are the *cf*-values (ratio of the number of correct residues to the total number of residues in the active site). For 1A6W in PDB two active sites (AS) are reported as HAP and AC1.

Here, we give a summary discussion regarding the properties of the unliganded protein structure 1A6U. It has 1737 atoms and its box has 43 × 49 × 41 voxels. The convex hull completely surrounded by triangles involves 148 triangles, which means the 1A6U structure can have at most 148 possible pockets. However, only 81 pockets remain with a 0.8 overlap cutoff between pockets. By using biochemical conditions, only 20 pockets remain and then by using physical conditions of depth and surface, only 16 pockets remain. These remaining pockets are listed in Table 3. Finally, only four pockets are left with a *cf* of 25% correctly predicted residues as shown in Table 4. The liganded protein reported in the PDB is 1A6W (1774 non-water atoms), and has the NIP ligand, which has 17 atoms with an 8.97 Å length and a 20.87 Å^2^ surface area. Thus, the protein pockets should have values of depth and surface area greater than these. The minimum distance between the atoms of ARG 350H in 1A6U with the atoms of the active sites in 1A6W is 2.89 Å. Table 4 shows the pockets’ residues and their minimum residue distances for 1A6U to the ligand atoms of NIP reported in the heterogenic atom lines in the PDB file of 1A6W.

**Table 4:** 1A6U best pockets with residues in common with the 2 active sites, HAP and AC1


**POCKET # 1, *cf*** **= 0.31 & 0.33**		
ASN 354H (11.61)	SER 331H (10.79)	TYR 34 L (4.27)
ASP 352H (7.07)	THR 328H (14.41)	TYR 332H (8.34)
ILE 351H (6.25)	THR 330H (12.29)	TYR 401H (2.92)
SER 32 L (6.81)	TRP 333H (1.734)	TYR 402H (5.75)
**POCKET # 39, *cf*** **= 0.31 & 0.22**		
ALA 2 L (15.1365)	HIS 97 L (6.8477)	THR 26 L (15.7431)
ARG 350H (2.89)	ILE 348H (9.34)	TRP 98 L (3.24)
ASN 96 L (7.12)	LYS 359H (5.38)	TRP 347H (4.78)
ASN 361H (9.75)	LYS 365H (14.84)	TYR 94 L (7.84)
GLU 362H (12.30)	PHE 364H (13.46)	TYR 360H (8.34)
GLY 349H (6.45)	SER 366H (17.38)	VAL 99 L (9.69)
**POCKET # 137, *cf*** **= 0.25 & 0.33**		
ASP 400H (5.44)	THR 31 L (8.29)	TYR 401H (2.92)
SER 405H (3.65)	TYR 34 L (4.27)	TYR 402H (5.75)
**POCKET # 143, *cf*** **= 0.44 & 0.56**		
ARG 350H (2.89)	SER 95 L (5.42)	TYR 332H (8.34)
ASN 354H (11.61)	SER 331H (10.79)	TYR 401H (2.92)
ASP 352H (7.07)	TRP 93 L (3.36)	TYR 402H (5.75)
ILE 351H (6.25)	TRP 333H (1.73)	
SER 32 L (6.81)	TYR 34 L (4.27)	

There are four predicted pockets with more than 25% of residues in common between the pockets and the active sites. The values in parentheses are the minimum residue distances for 1A6U to the ligand atoms of NIP reported in the heterogenic atom lines in the PDB file of 1A6W.

Table 3 gives all pockets of 1A6U, where only the two last columns are obtained by the comparison of the results with the binding sites HAP and AC1 of 1A6W (the corresponding liganded protein of 1A6U). In Table 3 the pockets are numbered and ordered arbitrarily. This table and all results were produced independently of the final answer.

**Figure 6:**
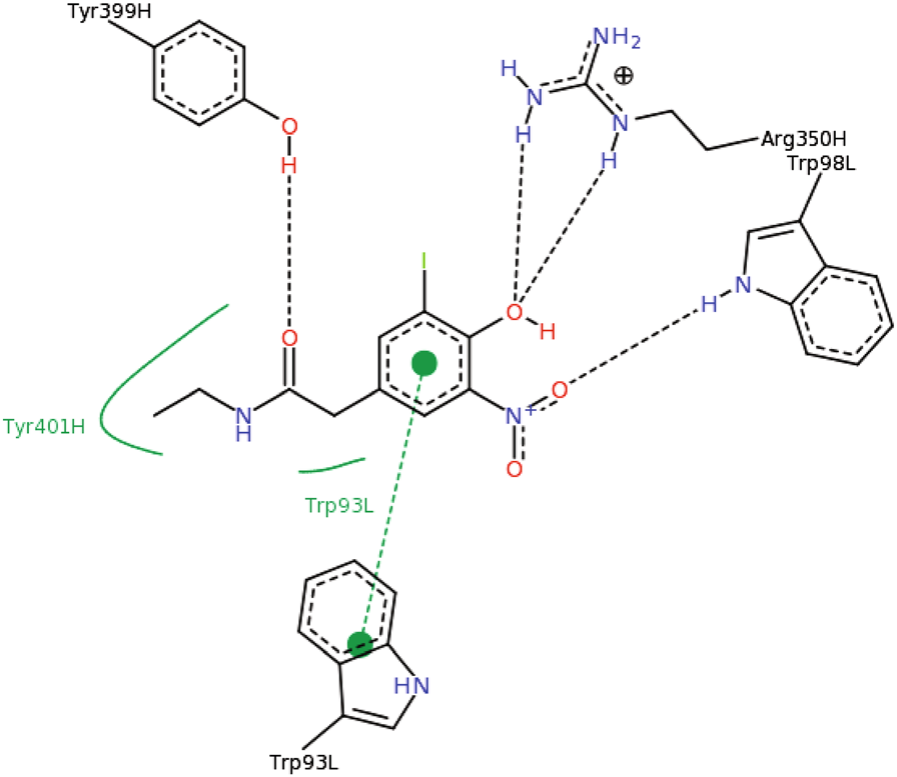
**1A6W and its ligand.** From the PDB website.

As can be seen in Figure 6, which is shown in the PDB website for the 1A6W protein, only five residues – TYR 399H, ARG 350H, TRP 93 L, TYR 401H and TRP 98 L – participate in the interaction with the NIP ligand, while in the PDB file of 1AW6 two active sites with 16 and 10 residues are reported (using the SITE REMARK lines in the PDB file). This shows that a maximum of 50% of the active site residues reported in the PDB for 1A6W participate in the interaction with the NIP ligand (a *cf* equal to 0.5). In our computation, for example, in the unliganded protein 1A6U the best pocket has a *cf* equal to 0.43 and to 0.55 for the first and second active site of the liganded protein 1A6W, respectively.

For illustration purposes we have taken the set of 48 and 86 “liganded and unliganded proteins”, respectively, listed in the supplementary material of Li et al. [20] and downloaded the files from the PDB site (see Additional file 1 for a list of the PDB files). We found the pockets of the *unliganded proteins*, and then we compared these pockets with the known active sites reported in the PDB files of the corresponding *liganded proteins*.

**Figure 7:**
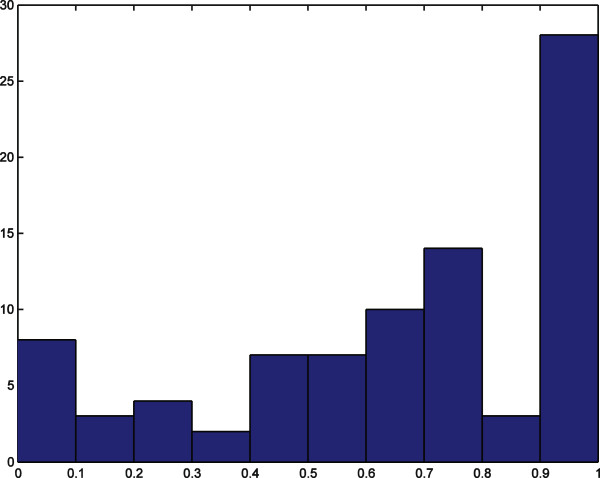
**Histogram of the 86-element data set.** Due to the RAM memory limits the protein number 55 in the 86-element data set list (PDB structures 2NGR and 1KZ7) was not included. The results are reported for the 85-element data set. The horizontal axis is the percentage of correct prediction of residues. The vertical axis is the number of proteins. The number of proteins with predicted pockets including more than half of the active site residues is 66 proteins (78% of the data set). Overlap threshold between pockets is 0.8.

**Figure 8:**
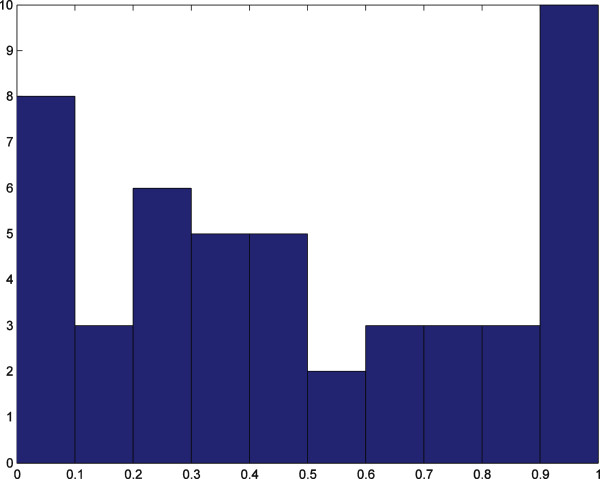
**Histogram of the 48-element data set.** The horizontal axis is the percentage of correct prediction of residues. The vertical axis is the number of proteins. The number of proteins with predicted pockets including more than half of the active site residues is 24 proteins (50% of the data set). Overlap threshold between pockets is 0.8.

The correct fraction, *cf*, of residues predicted in a given pocket is computed and the histograms of maximum *cf* in each protein’s pockets are reported in Figures 7 and 8. These results are obtained for a 0.8 overlap cutoff between pockets, and they show that 76% of the pockets predicted by our algorithm in the 86-element data set have at least half of their residues belonging to an active site in the liganded protein; for the 48-element data set the corresponding number is 50%. By using instead a 0.5 overlap cutoff, the results are 78% and 54% for the 86-element and the 48-element data set, respectively. Note that not all residues in the active sites reported in the PDB participate in protein-ligand interactions.

In Table 5 we compare the performance of our method with the other methods CASTp, LIGSITE, PASS, SURFNET and VISGRID. This table shows that our method with an overlap cutoff of 0.8 has comparable performance with the other methods. We should also note that the low computational cost of our method is a major advantage. In Additional file 2, full pockets of the 48-element set with a *cf* (ratio of the number of correct residues to the total number of residues in the active site) of more than 25% are reported. Additional file 2 also gives the minimum distance between each residue of the protein and ligand atoms.

**Table 5:** Performance comparison of our results with the other methods CASTp, LIGSITE, PASS, SURFNET and VISGRID

	**48 Unbound structures**	**86 Unbound structures**
	**(Top 1)**	**(Top 1)**
CAST	31 (64.6%)	66 (76.7%)
LIGSITE	36 (75.0%)	69 (80.2%)
PASS	27 (56.3%)	54 (62.8%)
SURFNET	19 (39.6%)	63 (73.3%)
VISGRID: Top 0.8% voxels	34 (70.8%)	55 (64.0%)
Our method: Overlap 0.8	24 (50%)	66 (78%)

The other results reported in Table III of Li et al. [20].

**Figure 9:**
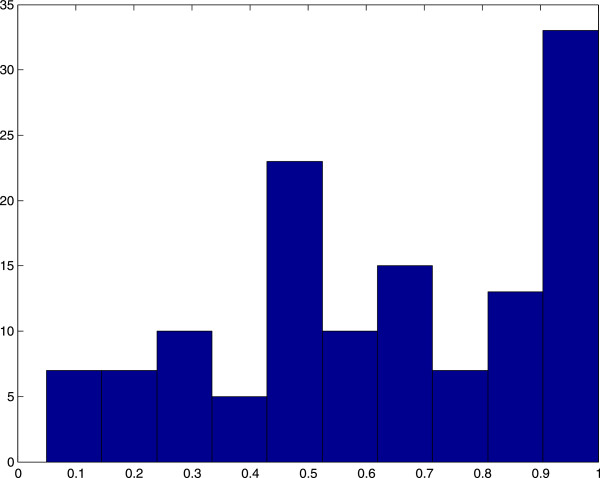
**Histogram of the 130-element data set.** The horizontal axis is the percentage of correct prediction of residues. The vertical axis is the number of proteins. Overlap threshold between pockets is 0.8.

We have also chosen another 130 pairs of unliganded and liganded protein structures of (listed in Additonal file 3). In Figure 9 the histograms of the maximum *cf* in each protein’s pockets are reported (with a 0.8 overlap). It shows that 73.8% of the pockets predicted by our algorithm in the 130-element data set have at least half of their residues belonging to an active site in the liganded protein, i.e. *cf* ≥ 0.5.

An important step which allows a decrease of the time and effort for the drug discovery process is to find suitable ligands through *in silico* methods using, for example, the virtual screening techniques. Our algorithm is easy to use and the cost of computation is approximately between 10 seconds for small proteins and up to 320 seconds for large proteins. The program was implemented in Matlab. The computer used for these computations is a laptop with an Intel Core i7 CPU and 8 GB RAM. The program usually uses 13% of the CPU time, but sometimes for a while it uses up to 50%. The program also while occupied in computation usually required less than 0.5 GB of RAM memory, but it was observed for some proteins to go up to 2 GB. The execution time for the 130 pair dataset is given in Additional file 3.

## Conclusions

In this paper, we have introduced a new simple method for predicting putative ligand-binding protein pockets. For each pocket, we can identify possible interacting protein atoms and residues, surface atoms, and also determine the size of a pocket (volume, surface area and depth). This information can help us verify possible ligands having a shape and size that is geometrically compatible with the pocket, and thus could be docked to the protein. We have used some biochemical properties to find the possible interacting atoms and residues in the pockets. Our method is a low cost computational method which voxelizes the protein space, and uses the convex hull concept commonly employed in computational geometry. This method could be used to classify proteins by the geometric properties of their pockets and also by their biochemical properties. An application of this method could be useful in reducing the cost and time of drug discovery.

## Competing interests

The authors declare that they have no competing interests.

## Authors’ contributions

SMSF and JAT conceived of the study, and participated in its design and coordination and helped to draft the manuscript. Both authors read and approved the final manuscript.

## Supplementary Material

Additional file 1 **Table with pairs (bound and unbound) of PDB files in the 48 element set and in the 86 element set.** For each pair, the RMSD (in angstroms) is given. Click here for file

Additional file 2 **List of the full pockets for each unliganded structure in the 48-element set with a *cf *** **(ratio of the number of correct residues to the total number of residues in an active site) of more than 25%.** For each pocket the *cf* for each active site (“AC”) is given after the label “Res. in common with *N* AC:”. Residues are named in the form “<resname > <resid > <chain>”. For each residue in a pocket, the minimum distance between the residue and the ligand atoms of the corresponding liganded structure is given. Click here for file

Additional file 3 **Tab-delimited text file.** Table with 130 pairs of unliganded (unlig) and liganded (lig) PDB files. For each pair, the *cf* and the time of execution (in seconds) is given. Click here for file
